# Detection of azole-resistant *Aspergillus fumigatus* in the environment from air, plant debris, compost, and soil

**DOI:** 10.1371/journal.pone.0282499

**Published:** 2023-03-03

**Authors:** Luisa F. Gómez Londoño, Marin T. Brewer

**Affiliations:** Department of Plant Pathology, University of Georgia, Athens, Georgia, United States of America; University of Tsukuba, JAPAN

## Abstract

*Aspergillus fumigatus* is a ubiquitous fungus, a saprophyte of plants, and an opportunistic pathogen of humans. Azole fungicides are used in agriculture to control plant pathogens, and azoles are also used as a first line of treatment for aspergillosis. The continued exposure of *A*. *fumigatus* to azoles in the environment has likely led to azole resistance in the clinic where infections result in high levels of mortality. Pan-azole resistance in environmental isolates is most often associated with tandem-repeat (TR) mutations containing 34 or 46 nucleotides in the *cyp51A* gene. Because the rapid detection of resistance is important for public health, PCR-based techniques have been developed to detect TR mutations in clinical samples. We are interested in identifying agricultural environments conducive to resistance development, but environmental surveillance of resistance has focused on labor-intensive isolation of the fungus followed by screening for resistance. Our goal was to develop assays for the rapid detection of pan-azole-resistant *A*. *fumigatus* directly from air, plants, compost, and soil samples. To accomplish this, we optimized DNA extractions for air filters, soil, compost, and plant debris and standardized two nested-PCR assays targeting the TR mutations. Sensitivity and specificity of the assays were tested using *A*. *fumigatus* DNA from wild type and TR-based resistant isolates and with soil and air filters spiked with conidia of the same isolates. The nested-PCR assays were sensitive to 5 fg and specific to *A*. *fumigatus* without cross-reaction with DNA from other soil microorganisms. Environmental samples from agricultural settings in Georgia, USA were sampled and tested. The TR_46_ allele was recovered from 30% of samples, including air, soil and plant debris samples from compost, hibiscus and hemp. These assays allow rapid surveillance of resistant isolates directly from environmental samples improving our identification of hotspots of azole-resistant *A*. *fumigatus*.

## Introduction

*Aspergillus fumigatus* is a ubiquitous fungus with a worldwide distribution. It is thermotolerant and thrives on decomposing organic matter making compost and decaying plants hotspots for its development and reproduction, and reservoirs of this fungus in the environment [1, 2]. *A*. *fumigatus* is an opportunistic human pathogen that causes environmentally-acquired aspergillosis in both immunocompromised and non-immunocompromised patients, from mild forms like allergies to lethal forms like invasive aspergillosis (IA) [1–3]. The most affected groups are those with cystic fibrosis, hematological malignancies, and recipients of organ transplants. The first line of treatment for aspergillosis is azole antifungals, including voriconazole, itraconazole, and posaconazole; however, azole-resistant *A*. *fumigatus* isolates are leading to treatment failures and have been detected in increasing amounts in patients around the world [4–6].

The two routes of azole resistance development in *A*. *fumigatus*–clinical or environmental–are based on the source of antifungal exposure. The first route occurs when resistant isolates evolve in patients undergoing treatment with azoles. The second route occurs in resistant isolates in azole-naïve patients that evolved resistance in the environment, likely from agricultural azole use [4–9]. Tebuconazole and propiconazole are the most readily available and used azoles to prevent fungal diseases in crops. These fungicides are structurally similar to clinical azoles and all azoles target the enzyme 14-alpha demethylase, also known as ERG11 or Cyp51A in some fungi. Loss of activity of Cyp51A in *A*. *fumigatus* interrupts ergosterol biosynthesis leading to the accumulation of demethylated intermediate toxic sterols increasing the permeability of the membrane and stopping fungal growth [6]. Several mutations in the *cyp51A* gene have provided *A*. *fumigatus* with azole resistance [4, 5]. The most frequently detected resistance alleles in *cyp51A* associated with the environmental route of resistance in *A*. *fumigatus* are tandem repeats (TR) in the promoter associated with non-synonymous point mutations in the coding region: TR_34_/L98H and TR_46_/Y121F/T298A [4, 6, 10, 11]. These two alleles in *A*. *fumigatus* confer pan-azole resistance, which is defined as a high level of resistance to multiple azole antifungals [8, 12].

Studies on antifungal-resistant *A*. *fumigatus* in the environment traditionally rely on time-consuming and labor-intensive methods. First, the fungus is isolated from environmental samples onto agar medium with components that may include Rose Bengal and antibiotics, followed by incubation at higher temperatures (>37°C) to minimize the growth of other microorganisms, including yeast, other fungi, and bacteria [9, 12]. Isolation is followed by antifungal resistance evaluations, such as inoculations onto antifungal-amended media [13] or minimum inhibitory concentration (MIC) assays according to the susceptibility testing guidelines of the European Committee on Antimicrobial Susceptibility Testing (EUCAST) or the Clinical Laboratory Standards Institute (CLSI) [5, 14, 15]. Often, the molecular mechanism of azole resistance is investigated in resistant isolates by extracting DNA and sequencing the *cyp51A* gene and its promoter region to identify if alleles associated with azole resistance are present [10, 16, 17].

Due to the time consuming and labor intensive methods for detection of azole-resistant isolates, molecular techniques were developed for rapid detection of azole-resistant *A*. *fumigatus* strains directly from clinical samples [18–21]. Rapid detection of azole-resistant *A*. *fumigatus* in environmental samples would help in environmental surveillance efforts aimed at identifying hotspots and understanding where azole resistance is most likely to evolve and propagate. To our knowledge, direct assessment of azole-resistant *A*. *fumigatus* in environmental samples using molecular assays has not been previously reported. Here, we describe standardized and rapid molecular methods to detect pan-azole-resistant *A*. *fumigatus* directly from air, soil, compost, and plant debris. We test the methods on environmental samples from Georgia, USA to identify sites with resistant *A*. *fumigatus* and to determine the resistance allele(s) present.

## Materials and methods

### Optimization of nested PCR assays to detect *A*. *fumigatus* in environmental samples

Three *A*. *fumigatus* isolates from the environment with different *cyp51A* alleles were selected to optimize the nested PCR assays. The resistance phenotypes and genotypes were described previously [12]. Isolate 1205 is an azole-sensitive, wild-type (wt) isolate without a tandem repeat (TR) mutation in the promotor of *cyp51A* and the same allele as reference strain A1163, whereas isolate 1467 has the TR_34_/L98H allele, and isolate 1470 has the TR_46_/Y121F/T298A allele. Isolates were maintained on Sabouraud dextrose agar (MilliporeSigma, Burlington, MA, USA) at 25°C prior to use. For DNA extraction, the isolates were grown in 30 ml flat bottom culture tube slants with potato dextrose agar (PDA, BD, Franklin Lakes, NJ, USA). After 1 week, 1 ml of saline solution containing 0.85% sodium chloride (Thermo Fisher Scientific, Pittsburgh, PA, USA), 0.05% Tween 20 (Thermo Fisher Scientific), and 0.1% gentamycin at 50 mg/ml (Thermo Fisher Scientific) was added to the tube. The suspension was vortexed briefly and approximately 800 μl of the saline solution and suspended fungal tissue were transferred to the 2 ml disruption tube provided with the Qiagen DNeasy Plant Pro kit (QIAGEN, Germantown, MD, USA) for DNA extraction following the manufacturer’s instructions. DNA was quantified with a NanoDrop One (Thermo Fisher Scientific) at concentrations greater than 2 ng/μl and diluted to a concentration of 1 ng/μl for PCR.

Two nested PCR assays originally developed for diagnosis and detection of TR_34_ (TR34-PCR) and TR_46_ (TR46-PCR) alleles in the promoter of *cyp51A* in *A*. *fumigatus* in clinical samples [15, 16] were evaluated for environmental detection of pan-azole-resistance. We tested both assays to determine if resistance alleles were preferentially amplified and if one worked better than the other for different environmental samples since they were designed for clinical samples. Each nested PCR assay uses two sets of primers. The TR34-PCR [15] uses the outer primers CypA-TR-S1 (5’-GGA GAA GGA AAG AAG CAC TCT-3’) and CypA-TR-AS1 (5’-TCA CCT ACC TAC CAA TAT AGG-3’) and the inner primers CypA-TR-S_A (5’-AGC ACC ACT TCA GAG TTG TCT A-3)’ and CypA-TR-AS_A (5’-TGT ATG GTA TGC TGG AAC TAC ACC TT-3’). The external primers amplify a 235-bp fragment within which the internal primers amplify a 100-bp fragment for isolates without a TR in the promoter. The TR46-PCR [16] uses the outer primers TR46long-S (5’-AAG CAC TCT GAA TAA TTT ACA-3’) and TR46long-AS (5’-ACC AAT ATA GGT TCA TAG GT-3’) and the inner primers TR46short-S (5’-GAG TGA ATA ATC GCA GCA CC-3’) and TR46short-AS (5’-CTG GAA CTA CAC CTT AGT AAT T-3’). The external primers amplify a 240-bp fragment within which the internal primers amplify a 103-bp fragment for isolates without a TR in the promoter. Primers were synthesized by Genewiz (South Plainfield, NJ, USA). Both assays were standardized for laboratory conditions by testing a gradient of annealing temperatures for the inner and outer primers at 52°C +/- 5°C.

The standardized PCR conditions for the nested TR34-PCR and TR46-PCR were the same for both assays. The first round of PCR contained 1 μl of the DNA (1 ng) in a final volume of 25 μl per reaction with 12.5 μl OneTaq HS Quick Load PCR mix (BioLabs, Ipswich, MA, USA) and a final concentration of 0.2 mM of each outer primer. The nested PCR was similar except 2 μL of PCR product from the first round of PCR and 0.2 mM of each inner primer was used. The temperatures and times for the first reaction were as follows: one cycle at 94°C for 2 min; 23 cycles at 94°C for 45 s, 52°C for 1 min, and 72°C for 1 min; and a final cycle at 72°C for 5 min. The second reaction consisted of one cycle at 94°C for 2 min, 35 cycles at 94°C for 45 s, 56°C for 1 min, and 72°C for 1 min, and a final extension at 72°C for 5 min.

Electrophoresis was conducted on a 2.5% agarose D1-LE (Alfa Aesar, Haverhill, MA, USA) gel to distinguish fragments with no TR from TR_34_ or TR_46_ alleles in the nested PCR amplification products. The agarose was amended with 1X GelRed (Biotum, Fremont, CA, USA) prior to casting for DNA visualization by UV light. Electrophoresis was performed for 90 min at 90 V with 5 μl of the PCR product in 2 μl of loading buffer.

### Detection limit of the nested PCR assays TR34-PCR and TR46-PCR

The detection limit was defined as the least amount of *A*. *fumigatus* DNA or conidia that could be detected and visualized by electrophoresis on an agarose gel using different extraction methods and the two nested PCR assays. The detection limit was tested directly on *A*. *fumigatus* DNA of known quantities and on extracted DNA from soil spiked with known quantities of conidia. Detection of *A*. *fumigatus* on air filters was tested by spiking filters with a known quantity of conidia. The DNA of isolates 1205 (no TR), 1467 (TR_34_), and 1470 (TR_46_) were diluted to concentrations of 50, 40, 30, 10, 5, 2, and 1 fg/μl by using the Qubit 2.0 Fluorometer (Invitrogen, Waltham, MA, USA) and Qubit dsDNA HS Assay Kit which is accurate for DNA quantification as low as 100 pg. Both nested PCR assays were conducted as described above with 1 μl of each DNA dilution. Conidial suspensions of *A*. *fumigatus* isolates 1205 (no TR), 1467 (TR_34_), and 1470 (TR_46_) were prepared at concentrations containing 10, 30, 60, 80, and 100 conidia per ml in the saline solution described above. Soil collected from farms in Georgia was autoclaved in a glass container wrapped in autoclave paper for 30 min. Soil was autoclaved to compare with non-autoclaved soil to ensure organisms and inhibitors were not interfering with the nested PCR assays. Five grams of autoclaved and non-autoclaved soil samples were portioned into 50-ml sterile Falcon tubes (Corning Life Sciences, Corning, NY, USA). The samples were mixed with 45 ml (1/10 w/v) of each conidial suspension. To verify that the filter did not inhibit PCR, 70-mm Whatman #6 cellulose filters (Cytiva Life Sciences, Marlborough, MA, USA) used for air sampling were cut in half (approximately 0.2 g), added to 15-ml sterile Falcon tubes, and inoculated with 1.8 ml (1/10 w/v) of 10,000 conidia per ml conidial suspensions of *A*. *fumigatus* isolates 1205 (no TR), 1467 (TR_34_), and 1470 (TR_46_). DNA was extracted using the flotation method and commercial extraction kits as previously described [22]. The flotation method allows for the recovery and concentration of fungal spores and hyphae from environmental samples. The samples in saline suspensions were mixed vigorously with a vortex for 1 min at maximum speed and allowed to settle for 20 min. This procedure was repeated, then the samples were vortexed for 1 min for a third time. After the largest particles had settled (approximately 5 min), 500 μl of the supernatant, which would contain the conidia and hyphal fragments, was collected for DNA extraction. The DNeasy PowerSoil Kit (QIAGEN) was used to extract DNA from soil samples and the DNeasy Plant Pro Kit (QIAGEN) was used to extract DNA from filters. Both kits were used according to the manufacturer’s instructions. The DNA was quantified by NanoDrop One with a final concentration of approximately 2 ng/μl, which is the detection limit for quantification. The two nested PCR assays were conducted as described above with 5 μl template DNA per reaction. Three positive controls–*A*. *fumigatus* isolates 1205 (no TR) and 1467 (TR_34_) or 1470 (TR_46_) containing 1 μl of DNA (1 ng) as template–and one negative control containing 1 μl of sterile distilled water were included in the PCR assays. PCR products were separated by electrophoresis and visualized as described above.

### Specificity of the nested PCR assays

To determine if the nested PCR assays amplify only DNA of *A*. *fumigatus*, two evaluations were performed. First, the primers were tested *in silico* using the Primer3 platform (https://primer3.ut.ee/) and blastn from NCBI (https://blast.ncbi.nlm.nih.gov/). Each primer was blasted against sequences in the database to establish they paired only with *A*. *fumigatus*.

Next, both nested assays were tested with DNA of microorganisms commonly isolated from two soils samples from Georgia and Oregon, USA. The samples were prepared by adding a saline solution containing 0.85% sodium chloride and 0.05% Tween 20, followed by mixing with a vortex for 1 min at maximum speed and allowing to settle for 20 min. This procedure was repeated, then the samples were vortexed for 1 min for a third time. After the largest particles had settled (approximately 5 min), 500 μl of the supernatant was collected and serial dilutions of 1:10, 1:100, and 1:1000 were performed; from each dilution 200 μl was spread onto two plates of Sabouraud dextrose agar with and without gentamycin at final concentration of 0.05 mg/ml. The cultures were incubated at 25°C for 2 weeks and the Petri dishes were visually inspected on days 2, 5, 10, and 15 to recover different microorganisms. Each colony was sub-cultured in a Petri dish with SDA and identifications were based on morphological characteristics by microscopic observation with lactophenol blue or gram stain for fungi and bacteria, respectively. The microorganisms were grown in flat bottom glass culture tubes with PDA 1 to 2 weeks. Tissue was collected as described above for *A*. *fumigatus* and placed into the 2 ml disruption tube provided with the Qiagen DNeasy Plant Pro kit to undergo the DNA extraction following the manufacturer’s instructions. After DNA was obtained, 1 μl containing 1 ng/μl of each organism was used as template for the nested PCR assays with controls as described above.

### Collection of environmental samples

To test the assays on environmental samples and determine the proportion of *A*. *fumigatus* with and without the different TR alleles recovered from environments near Athens, Georgia, USA, thirty-three samples were collected from June to September of 2020 on the University of Georgia main campus in Athens, Georgia, the UGArden in Athens, Georgia, and the UGA Hort Farm in Watkinsville, Georgia. No permits were required to sample at these sites owned by the University of Georgia. The samples included 22 air samples, 5 soil samples, 2 plant debris samples and 4 compost samples (Table 1). To collect air samples, 1000 L of air was aspirated through 300 micropores in the head of a PCE-AS1 air sampler (PCE Holding GmbH, Hamburg, HH, Germany) at a nominal rate of 100 L per min at a height of 1 meter. The airflow was directed toward the 70-mm Whatman #6 cellulose filter surface placed inside a 100-mm Petri dish so that particles were deposited on the filter surface. Once air sampling was complete the lid was placed on the Petri dish with the filter inside. Soil and compost were collected using a soil sample probe. Plant debris on the ground was collected by hand. Approximately 100 g of soil, compost or plant debris was collected for each sample and placed into a 1-qt Ziploc bag. All samples were labeled and kept at 4°C until processing. DNA extraction was performed within one week of environmental sample collection.

**Table 1:** Environmental samples and TR34-PCR and TR46-PCR assay results.

Sample number	Sample type	Sampling date (YYYY-MM-DD)	Location	Substrate/crop	TR34-PCR^a^	TR46-PCR
1	Air	2020-06-17	UGA Campus	Lawn/Turf	-	-
2	Air	2020-06-17	UGA Campus	Lawn/Turf	-	-
3	Air	2020-06-24	UGA Campus	Lawn/Turf	-	-
4	Air	2020-06-24	UGA Campus	Lawn/Turf	-	-
5	Air	2020-06-24	UGA Campus	Lawn/Turf	-	-
6	Air	2020-06-24	UGA Campus	Lawn/Turf	+ wt	+ wt
7	Air	2020-06-24	UGA Campus	Lawn/Turf	+ wt	-
8	Air	2020-06-24	UGA Campus	Lawn/Turf	+ wt	+ wt
9	Air	2020-06-24	UGA Campus	Lawn/Turf	-	-
10	Air	2020-06-24	UGA Campus	Lawn/Turf	-	-
11	Air	2020-06-24	UGA Campus	Lawn/Turf	-	-
12	Air	2020-06-24	UGA Campus	Lawn/Turf	+ wt	-
13	Air	2020-06-24	UGA Campus	Lawn/Turf	-	+ wt
14	Air	2020-06-24	UGA Campus	Lawn/Turf	-	+ wt
15	Soil	2020-06-29	UGA Campus	Lawn/Turf	-	-
16	Soil	2020-06-29	UGA Campus	Lawn/Turf	+ wt	-
17	Soil	2020-06-29	UGA Campus	Lawn/Turf	-	-
18	Air	2020-09-04	UGArden	Compost	+ wt	+ TR46
19	Air	2020-09-04	UGArden	Compost	-	-
20	Compost	2020-09-04	UGArden	Compost	+ wt	+ wt
21	Compost	2020-09-04	UGArden	Compost	+ wt	+ wt
22	Air	2020-09-04	UGArden	Compost	-	-
23	Air	2020-09-04	UGArden	Compost	+TR46	-
24	Compost	2020-09-04	UGArden	Compost	-	-
25	Compost	2020-09-04	UGArden	Compost	-	-
26	Air	2020-09-04	Hort Farm	Hibiscus	+TR46	-
27	Air	2020-09-04	Hort Farm	Hibiscus	+TR46	-
28	Soil	2020-09-04	Hort Farm	Hibiscus	+TR46	-
29	Plant debris	2020-09-04	Hort Farm	Hibiscus	+TR46	-
30	Air	2020-09-04	Hort Farm	Hemp	+TR46	-
31	Air	2020-09-04	Hort Farm	Hemp	+TR46	-
32	Soil	2020-09-04	Hort Farm	Hemp	+TR46	-
33	Plant debris	2020-09-04	Hort Farm	Hemp	+TR46	-

^a^ “+ wt” indicates wild type *A*. *fumigatus* allele detected, “+TR46” indicates TR_46_ allele of *A*. *fumigatus* was detected, and “-” indicates no *A*. *fumigatus* alleles were detected.

### DNA extraction from environmental samples

The environmental samples were prepared for DNA extraction using the flotation method as described above for the inoculated soil and filter papers except the saline solution contained no added conidia. The extractions for compost were conducted the same as for soil, and the extractions for plant debris contained 2 g plant debris in 18 ml saline solution (1/10 w/v) in a 50-ml Falcon tube. The DNeasy PowerSoil Kit (QIAGEN, Germantown, MD, USA) was used to extract DNA from soil and compost samples and the DNeasy Plant Pro Kit (QIAGEN) was used to extract DNA from air filters and plant debris samples. Both kits were used according to the manufacturer’s instructions. The DNA extracted from the environmental samples was quantified by NanoDrop One with concentrations of approximately 2 ng/μl, which is the detection limit for quantification. To check the efficiency of the environmental DNA extractions, DNA integrity tests were conducted by running extracts on a 1% agarose electrophoresis gel and confirming the presence of high molecular weight bands without or with minimal smearing indicative of degraded DNA.

Environmental samples were tested in duplicate for both nested PCR assays as described above using 5 μl of each sample as template DNA in the PCR. The same controls were used as described above except the DNA was reduced to 0.2 ng for the positive controls. Because environmental samples such as soil and compost are known to have numerous PCR inhibitors, each environmental sample was run with an inhibition control. The inhibition control consisted of the addition of 2.5 μl of the DNA from the environmental sample and 2.5 μl DNA of the 1205 wild-type *A*. *fumigatus* isolate. Inhibition controls were run on a separate electrophoresis gel to avoid contamination. The absence of an amplification product in any of the inhibition controls would indicate the presence of PCR inhibitors. Inhibitors were never detected so further clean-up of the samples was not required and negative samples for detection of *A*. *fumigatus* could be considered true negatives.

Electrophoresis was conducted on a 2.5% agarose gel as described above to distinguish fragments with no TR from TR_34_ or TR_46_ alleles in the nested PCR amplification products of the environmental samples. When the larger fragments indicative of the TR_34_ or TR_46_ alleles were observed in the samples, the products were run on a gel with 4% of MethaPhor agarose (Lonza, Greenwood, SC, USA) to improve separation, visualization and identification of the specific TR allele. The agarose was amended with 1X GelRed (Biotum, Fremont, CA, USA) prior to casting for DNA visualization. Electrophoresis with 4% of agarose were performed for 5 h at 70 /V with 5 μl of the PCR product in 2 μl of loading buffer.

### Sequencing of the products amplified by the nested PCR assays TR34-PCR and TR46-PCR

To confirm that PCR products were of the *cyp51A* promoter region of *A*. *fumigatus* with or without tandem repeats, amplicons from the three *A*. *fumigatus* control isolates (1205/wt, 1467/TR_34_, and 1470/TR_46_) produced by the TR34-PCR and TR46-PCR assays were sequenced. Additionally, amplicons from environmental samples of 8 TR-positive and 4 non-TR *A*. *fumigatus* from the TR34-PCR assay and 4 wt positive from the TR46-PCR assay were sequenced. Amplicons were cut from agarose gels with a sterile scalpel and purified using the Qiagen II gel extraction kit (QIAGEN). Bidirectional Sanger sequencing of the amplification products using the internal primers of each PCR through the chain termination method was performed by GENEWIZ. The sequences were aligned and edited manually using Geneious Prime 2019.2.3 (San Diego, CA, USA). A multiple alignment was performed to observe the presence or absence of the tandem repeats. The Basic Local Alignment Search Tool (http://blast.ncbi.nlm.nih.gov/Blast.cgi) was used to verify that the sequenced PCR products matched *A*. *fumigatus*.

## Results

### Detection limits of the TR34-PCR and TR46-PCR assays

**Fig 1:**
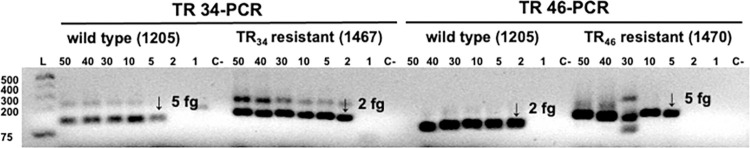
Detection limit of the TR34-PCR and TR46-PCR assays for *Aspergillus fumigatus*. The DNA from a wild type isolate (1205) and TR-based resistant isolates (1467 and 1470) were tested from 50 fg to 1 fg per reaction. The least amount of DNA detected is indicated with an arrow. Controls in the PCR assays included isolates 1205 (wt), 1467 (TR_34_), and 1470 (TR_46_) as positive controls and sterile distilled water as the negative control (c-).

The PCR products of the three *A*. *fumigatus* control isolates after the nested PCR were the expected sizes for both assays (Fig 1). For the TR34-PCR assay, the fragment size for the isolate without a TR in the promoter was 100 bp, whereas the fragment size for the isolates with TR_34_ was 134 bp. For the TR46-PCR assay, the fragment size for the isolate without a TR in the promoter was 103 bp, whereas the fragment size for the isolate with TR_46_ was 149 bp. The larger bands produced during the first round of PCR (235 bp and 240 bp for wt) in the TR34-PCR and TR46-PCR assays, respectively) were more visible in the TR34-PCR assay; however, as expected, the first-round PCR bands became fainter with decreasing amounts of template DNA. The quantity of *A*. *fumigatus* DNA detected using the TR34-PCR assay was 5 fg for the wt and 2 fg for the TR_34_ isolate. The detection limits for the TR46-PCR assay were 2 fg and 5 fg for the wt and the TR_46_ isolates, respectively (Fig 1). The theoretical detection limit was calculated using the average molecular weight of one nucleotide (618 g/mol), the number of nucleotides in the *A*. *fumigatus* genome (reference strain Af293, 29,420,142 bp), Avogadro’s constant, and the least amount of DNA detected by each PCR assay [22]. Since the molecular weight of one *A*. *fumigatus* genome was estimated to be 30.2 fg and given that the PCR assays detected in the fg level, the sensitivity and equivalent cell detection level for both nested PCR assays were a single cell of the fungus for both the wt and resistant isolates of *A*. *fumigatus*.

**Fig 2:**
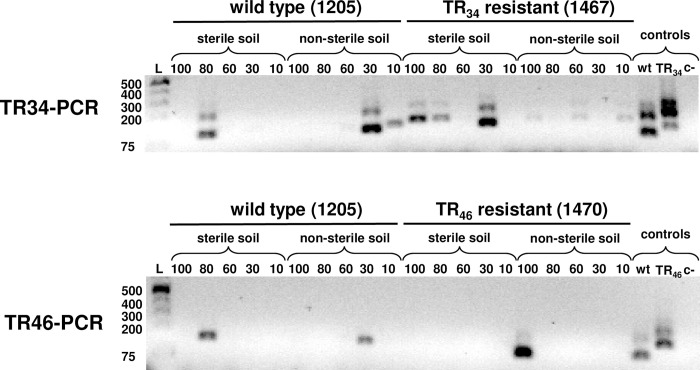
Sensitivity of the TR34-PCR and TR46-PCR assays. Assays were conducted for soil samples and autoclaved soil samples spiked with different quantities of conidia of *A*. *fumigatus*, including 100, 80, 60, 30, and 10 conidia per ml. The assays were also conducted on soil samples sterilized prior to spiking with conidia of *A*. *fumigatus*. Controls in the PCR assays included isolates 1205 (wt), and 1467 (TR_34_) or 1470 (TR_46_) as positive controls and sterile distilled water as the negative control (c-).

The sensitivity of both assays for detecting *A*. *fumigatus* in environmental samples was tested for soil samples and air filters spiked with conidia of the wt and resistant isolates. The concentrations of conidia per gram of soil ranged from 90 to 900 (45 ml of solution with 10 to 100 conidia per ml added to 5 g soil). Both PCR assays amplified the DNA of *A*. *fumigatus* directly from the aliquots of autoclaved and non-autoclaved soil spiked with conidia (Fig 2). Both wt and resistant isolates were detected by both assays using non-autoclaved soil. However, the detection of the DNA is inconsistent among the samples even though all were spiked with conidia. Based on this experiment, the sensitivity of the TR46-PCR assay was not good for soil (Fig 2). Both PCR assays could detect both wt and resistant *A*. *fumigatus* conidia from artificially spiked filter paper (S1 Fig).

### Specificity of the TR34-PCR and TR46-PCR assays

The in-silico analysis of the eight primers using the Primer3 platform and blastn, showed both assays bind only *A*. *fumigatus*. Three different types of bacteria (*Actinomyces* sp., *Bacillus* sp., coccobacilli) and eleven different types of fungi (*Candida* sp., *Penicillium* sp., *Chrysosporium* sp., *Fusarium* sp., *Paecilomyces* sp., *Rhizopus* sp., *Rhizomucor* sp., *Mucor* sp., *Cladosporium* sp., *Aspergillus flavus*, *Aspergillus niger*) were frequently recovered from two different soil samples from Georgia and identified based on their morphological characteristics. There was no amplification of DNA in the expected size ranges of PCR products from any of the microorganisms isolated from soil with the TR34-PCR or TR46-PCR assays (S2 Fig).

### Detection of azole-resistant *A*. *fumigatus* in environmental samples with the TR34-PCR and TR46-PCR assays

**Fig 3:**
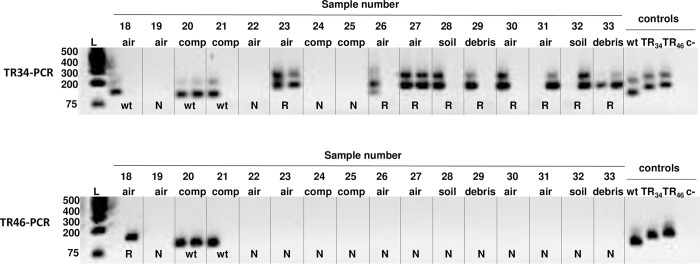
Gel image of 16 of the environmental samples, including air, compost (comp), soil, and plant debris (debris), assayed by TR34-PCR and TR46-PCR. Sample numbers above each lane correspond with samples in Table 1. DNA was extracted from each sample which was processed in duplicate for the nested PCR assays. Samples showing bands in one or both replicates were considered positive for *A*. *fumigatus*. The 2.5% agarose gel allows the differentiation of the wt allele from the TR_34_ and TR_46_ resistance alleles. Samples with the *A*. *fumigatus* wt allele for the *cyp51A* promoter yield PCR products of 100 bp and 103 bp for TR34-PCR and the TR46-PCR, respectively. Samples with the resistant alleles produce PCR products that are 134 or 146 bp for TR34-PCR or 137 or 149 bp for TR46-PCR. These samples are considered resistant (R) and undergo high-definition electrophoresis on a 4% gel to distinguish the TR_34_ and TR_46_ alleles. Samples without an amplification product were considered negative (N).

Both assays were conducted on thirty-three environmental samples that included twenty air samples, five soil samples, four plant debris samples, and four composted organic matter samples (Table 1). An example of the assays on several environmental samples and an interpretation of the results are shown (Fig 3).

**Fig 4:**
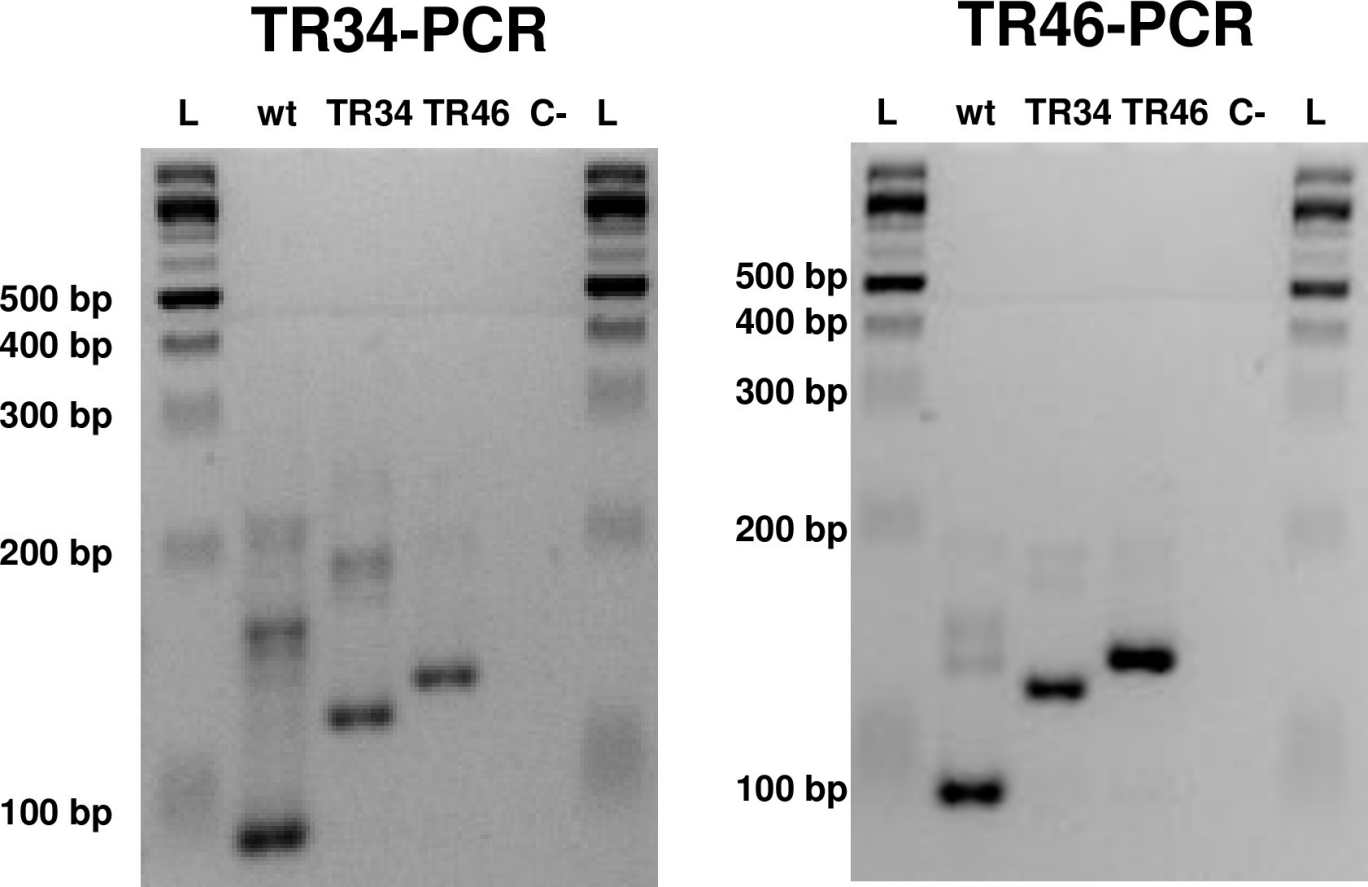
The nested TR34-PCR and TR46-PCR assays amplify *A*. *fumigatus* wild type (wt) alleles and the TR_34_ and TR_46_ alleles. Differentiation among these alleles can be accomplished with electrophoresis in 4% high-definition agarose.

**Fig 5:**
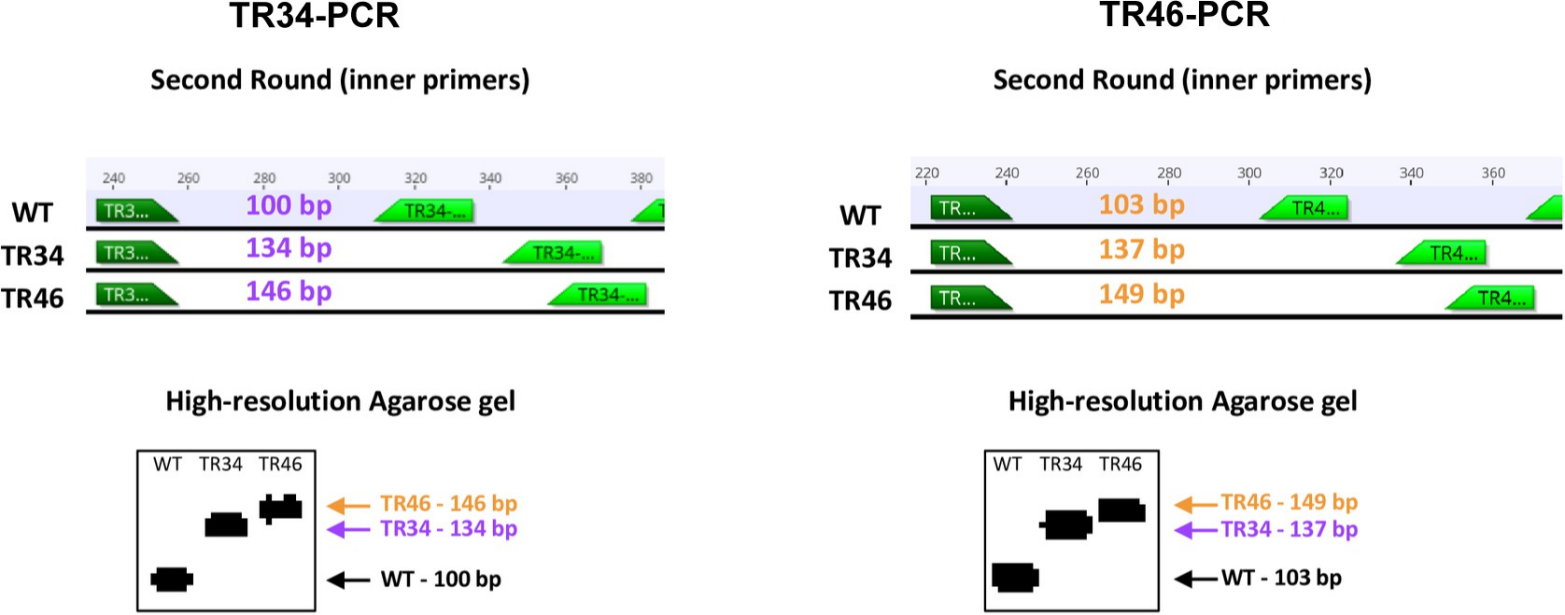
Fragment sizes of PCR products from the TR34-PCR and TR46-PCR assays. Sizes are shown for the *A*. *fumigatus cyp51A* wt, TR_46_, and TR_34_ alleles based on the nested or second round PCR with the inner primers.

Although the TR34-PCR and TR46-PCR were developed for detection of each of the corresponding resistance alleles [18, 19], both the TR_34_ and TR_46_ allele can be detected by both assays. If the nested PCR assays are followed by electrophoresis in 4% high-definition agarose the wt, TR_34_, and TR_46_ *A*. *fumigatus cyp51A* promoter alleles can be identified and differentiated in a single gel (Fig 4). For both the TR34-PCR and TR46-PCR assays, the fragment for the TR_46_ allele is 12 bp larger than the fragment for the TR_34_ allele, which is 34 bp larger than the fragment for the wt allele (Fig 5). The TR46-PCR assay detects the wt DNA better than either the TR34 or TR46 allele. The TR34-PCR assay better detects all three alleles.

With the TR34-PCR assay, we detected *A*. *fumigatus* in 51.5% (17 of 33) of the samples, and 27.3% (9 of 33) had a TR resistance allele (Table 1). Samples were considered positive for *A*. *fumigatus* if alleles were detected in only one of the replicates. One of the samples (no. 26) had both wt and TR resistance alleles (Fig 3) but was considered a resistant sample since the resistance allele was detected. With the TR46-PCR assay, we detected *A*. *fumigatus* in 21.2% (7 of 33) of the samples and one (3.0%) had the TR_46_ allele. Five samples (nos. 6, 8, 18, 20, and 21) were positive for wt *A*. *fumigatus* by both assays. Sample results were not always consistent between replicates or between assays. For example, sample nos. 30 and 31 each have one positive resistance allele (TR_46_) in one replicate for the TR-34 assay and are negative for the other replicate and are both negative for both replicates for the TR-46 assay (Fig 3). When the samples positive for a TR allele were electrophoresed using the high-definition 4% agarose it showed that these 10 samples had the TR_46_ allele. These included 8 air, soil, and plant debris samples from both hibiscus and hemp plots at the Hort Farm and two air samples near compost from the UGArden.

Since both wt and TR alleles could be detected in a single sample we wanted to make sure that all three alleles could be detected by the PCR-based assays in mixed samples. We ran both assays as described above with different proportions of control DNA and were able to detect all alleles present in the samples (S3 Fig).

To test if resistant isolates were present in samples that were positive for TR alleles, we tried culturing *A*. *fumigatus* from five positive soil samples on tebuconazole-amended (8 μg/ml) SDA [12]. We recovered tebuconazole-resistant *A*. *fumigatus* with TR_46_ alleles from sample #28 (hibiscus). We also recovered wt isolates of *A*. *fumigatus* from many of the wt-positive samples.

## Sequencing *cyp51A* alleles

To confirm that we were amplifying the alleles of the *A*. *fumigatus cyp51A* promoter region and that the resistance alleles were indeed TR_46_, PCR products of the positive controls from both assays, wt-positive sample nos. 6, 7, 8, and 12 and TR_46_-positive sample nos. 23, 27, 28, 29, 30, 31, 32, and 33 from TR34-PCR, and wt-positive sample nos. 6, 8, 13, and 14 from the TR46-PCR were sequenced. There was not enough DNA in the extracted band to sequence sample no. 18 from the TR46-PCR assay. We confirmed that the PCR products of the three positive controls were the expected alleles (Fig 5, S4 Fig). The PCR products from the wt environmental samples all had sequences similar to wt alleles and the sequences of the products from the samples that were the same size as the TR_46_ allele of the positive control (in the TR-34 assay) all had identical sequence to the TR_46_ allele. Sequences were confirmed as the corresponding alleles of *A*. *fumigatus* by BLASTn.

## Discussion

The TR34-PCR and TR46-PCR assays allowed us to rapidly detect azole-resistant *A*. *fumigatus* directly from environmental samples, including air, soil, compost, and plant debris, with high sensitivity and specificity similar to values reported for clinical samples [18, 19]. The ability to detect cells of *A*. *fumigatus* in more complex environmental samples such as soil and compost is critical since these samples are expected to have a richness of organisms, enzymes, and metabolites that increase the difficulty of detecting many microbes [23, 24]. We showed that both nested PCR assays allowed detection and discrimination of either of the two tandem repeat alleles, TR_34_ or TR_46_, from the wt allele when conducting electrophoresis using 1% agarose. The two *A*. *fumigatus* azole resistance alleles, TR_34_ or TR_46_, could be further distinguished from each other when using a high-resolution 4% agarose gel. Additionally, we were able to distinguish multiple alleles in the same sample (S3 Fig). Overall, the TR34-PCR assay was more sensitive, consistent, and reliable. A limitation of the assays is that there are false negatives. For example, we did not always detect resistant or wt isolates in PCR replicates of the more consistent and sensitive TR34-PCR assay (Fig 3). Multiple replicates are needed to best characterize *A*. *fumigatus* in samples. Although these PCR assays detect only the TR alleles associated with azole resistance, these alleles are by far the most common in the environment with 61% of environmental azole-resistant *A*. *fumigatus* having the TR_34_/L98H allele and 15% having the TR_46_/Y121F/T289A allele [11]. Moreover, these alleles are most often associated with both pan-azole resistance and high levels (MIC ≥ 16 μg/ml) of resistance to azole antifungals in both environmental and clinical isolates [8, 9, 11, 12, 14, 25]. Other TR resistance alleles, such as TR_53_ [26] and a triple repeat of TR_46_ [27], would likely be distinguishable from wt alleles with these PCR assays.

These nested PCR assays allow the identification of pan-azole-resistant isolates in a quarter of the time, or at least 6 days less, than standard microbiological techniques since the DNA extraction from the environmental samples, the nested PCR, and the high-resolution electrophoresis take approximately two days. The culturing of *A*. *fumigatus* from environmental samples, followed by MIC assays, DNA extraction, and sequencing of *cyp51A* to identify the genetic basis of resistance, takes from one to two weeks [28, 29]. Moreover, the PCR-based assays require far fewer resources, including both supplies and person hours. Far larger quantities of environmental samples can be rapidly screened for azole-resistant *A*. *fumigatus* using these assays making them a high throughput alternative to identifying hotspots and reservoirs of resistance. It is important to consider that with PCR-based detection assays we are detecting DNA and not necessarily living, viable organisms. If warranted, isolates of *A*. *fumigatus* could still be collected from samples of interest for additional studies such as whole genome sequencing and phenotyping assays, such as MIC.

In addition to soil, compost and plant debris samples, we were able to amplify DNA of *A*. *fumigatus* from cellulose filters with air impacted by a single-stage air sampler and identify if TR resistance mutations were present. Air sampling is critical for understanding transmission of azole resistant *A*. *fumigatus* since inhalation of the fungus in bioaerosols is the main route of infection [30]. Although *A*. *fumigatus* is a ubiquitous microorganism frequently reported as one of the top five fungal species present in bioaerosols [31], the lack of isolation of resistant isolates of *A*. *fumigatus* even with the use of different agar culture media and growth conditions has been discussed [29, 32]. Molecular diagnostic techniques based on PCR-based approaches overcome the problems related to microbiological isolation of *A*. *fumigatus* [29, 33].

Other methods have been developed for rapid detection of *A*. *fumigatus*, including real-time PCR [20] and loop-mediated isothermal amplification (LAMP) [34, 35]. These methods have been applied to clinical samples or to isolates in the case of LAMP. Here, we describe a rapid method for detection directly from environmental samples, such as soil, compost and air filters. The advantage to real-time PCR is that it is more sensitive, as well as quantitative, but it requires very expensive supplies and equipment, as well as highly trained personnel to interpret the results. Nevertheless, the development of a real-time PCR-based method for the direct detection and quantification of resistant *A*. *fumigatus* from environmental samples would be very useful. LAMP is a practical and easy method for rapid detection but the currently developed methods [34, 35] have not been tested on environmental samples. Moreover, in a single assay the nested PCR assays discriminates wt, TR34 and TR46 alleles, which are the two alleles most commonly associated with the usage of azoles in the agriculture [11]. On the other hand, the developed LAMP techniques exclusively detect the TR34 [34] or TR46 [35] alleles.

When we used the nested PCR assays to detect azole-resistant *A*. *fumigatus* from environmental samples collected in the Athens and Watkinsville, Georgia, we found the TR_46_ allele in 30.3% of the environmental samples. We did not detect the TR_34_ allele from environmental samples in this study. Eight of the samples positive for azole-resistant *A*. *fumigatus* came from the Hort Farm where azole fungicides are widely used to control plant-pathogenic fungi. Additionally, flower farms are a known hotspot for azole resistance in the environment [11] and our detection of the resistance allele in air, soil, and plant debris in a hibiscus field further supports this. We were able to recover azole-resistant *A*. *fumigatus* isolates with the TR46 allele from hibiscus soil samples. Additional studies of soil, air, and debris from hemp fields are necessary to determine if hemp is a potential hotspot for azole-resistant *A*. *fumigatus*. The other two TR_46_ positive results in this study came from an air sample collected near compost, which is a known hotspot for azole-resistant *A*. *fumigatus* [36]. Though the TR_34_ allele is most frequently associated with azole resistance in agricultural environments [11], a previous study detected 13 pan-azole-resistant isolates from compost and pecan debris in Georgia, all with the TR_46_ allele [12]. Another study of an agricultural site in Georgia detected 20 azole-resistant isolates all with TR_34_ alleles in a peanut debris pile [15]. More extensive sampling of the environment is required to determine the relative frequencies of these resistance alleles in isolates in Georgia, and to determine the impact their abundance has on disease transmission, antifungal resistance and human health.

Here, we validated the use of two molecular assays based on nested PCR that allow the simultaneous detection and characterization of the tandem repeat alleles in *A*. *fumigatus* associated with resistance to azoles directly in environmental samples. These rapid, sensitive, and specific assays will improve our understanding of azole resistance development and persistence of *A*. *fumigatus* in agricultural environments.

## Supporting information

S1 Fig Detection of *Aspergillus fumigatus* from filter paper spiked with conidia for the TR34-PCR and TR46-PCR assays. L: Ladder, lane 1: wt *A*. *fumigatus*, lane 2: TR_34_ *A*. *fumigatus*, lane 3: negative control, lane 4: empty, lane 5: wt *A*. *fumigatus*, lane 6: TR_46_ *A*. *fumigatus*, lane 7: negative control.
(TIF)

S2 Fig Specificity of TR34-PCR and TR46-PCR assays for *Aspergillus fumigatus*. There was no amplification of DNA for any of the other microorganisms tested. There were amplification products of the wt, TR_34_, and TR_46_ *A*. *fumigatus* isolates. L: Ladder, lane 1: Actinomycete, 2: *Bacillus* sp., 3: Coccobacillus, 4: *Candida* sp., 5: *Penicillium* sp., 6: *Chrysosporium* sp., 7: *Fusarium* sp., 8: *Paecilomyces* sp., 9: *Rhizopus* sp., 10: *Rhizomucor* sp., 11: *Mucor* sp., 12: *Cladosporium* sp., 13: *Aspergillus flavus*, 14: *Aspergillus niger*, wt: wild-type *A*. *fumigatus* isolate, TR34: *A*. *fumigatus* isolate with the 34-nucleotide tandem repeat, TR46: *A*. *fumigatus* isolate with the 46-nucleotide tandem repeat.
(TIF)

S3 Fig Nested PCR assay performance with mixtures of alleles. Both TR34-PCR and TR46-PCR amplify all alleles in mixtures. Here we mixed DNA from *Aspergillus fumigatus* controls with wt, TR_34_ and TR_46_ alleles in different combinations. DNA was mixed in equal proportions with final DNA concentrations of 0.2 ng/μl. It is possible to detect and distinguish the three alleles in various mixtures in a 4% agarose gel by comparing the fragment sizes in the mixtures with the fragments from the samples with single alleles. The bands in mixtures are fainter because the alleles are represented by either half or one third as much DNA as the single isolate samples in the template DNA used for PCR.
(TIF)

S4 Fig Sequence alignment from the TR34-PCR and TR46-PCR assays. The PCR products from the environmental samples in Athens and Watkinsville, GA were confirmed as having the wt or TR_46_ alleles. Nucleotide lengths of sequences for control isolate alleles corresponded with the fragment sizes on the agarose gels.
(TIF)

S1 Raw images (PDF)
