# Supplementary figures and images for: Detection of azole-resistant Aspergillus fumigatus in the environment from air, plant debris, compost, and soil
Source: PLoS One. 2023 Mar 3;18(3):e0282499. doi: 10.1371/journal.pone.0282499 (PMC9983824; doi:10.1371/journal.pone.0282499)

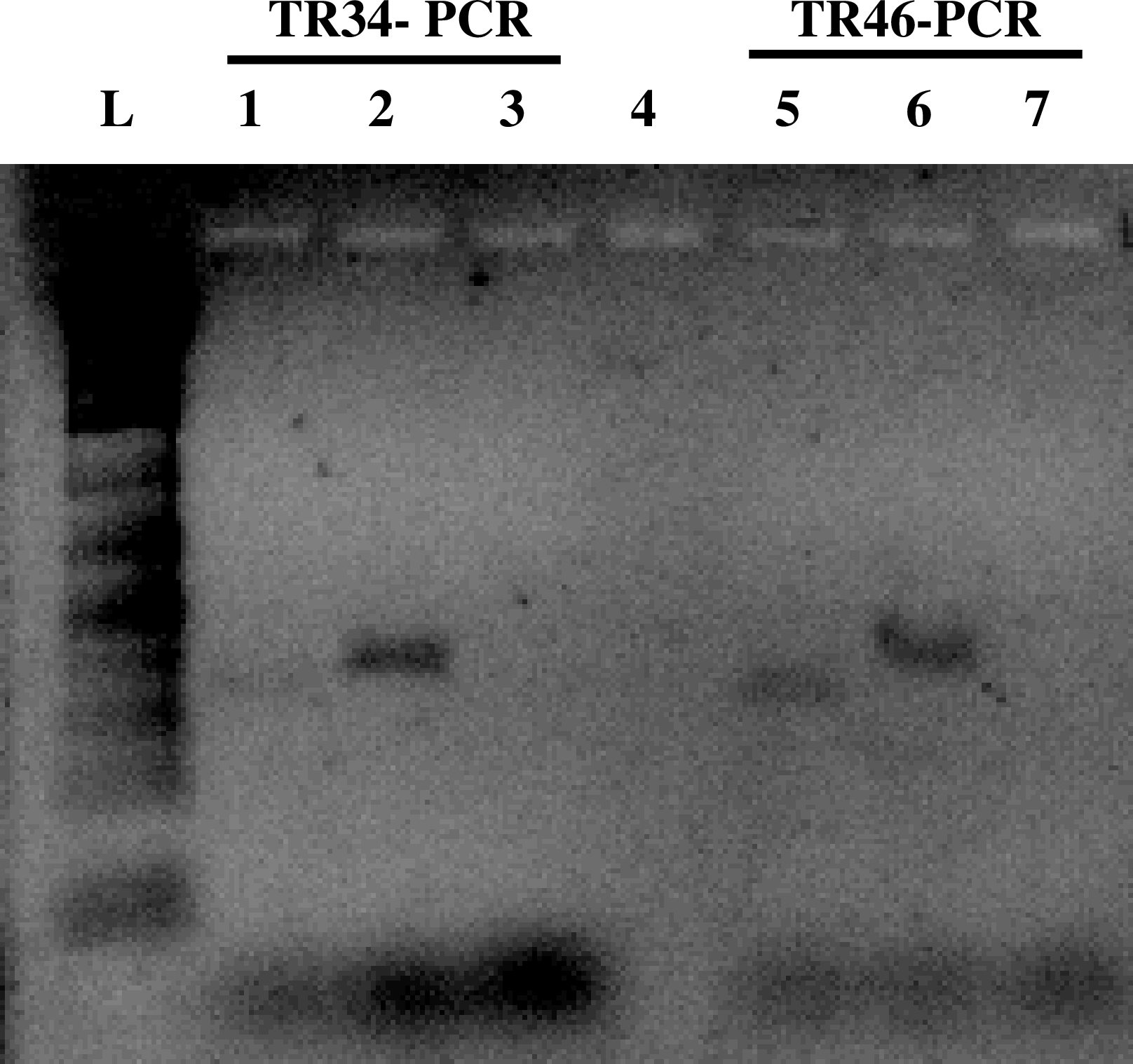

Supplement: S1 Fig — L: Ladder, lane 1: wt A. fumigatus, lane 2: TR34 A. fumigatus, lane 3: negative control, lane 4: empty, lane 5: wt A. fumigatus, lane 6: TR46 A. fumigatus, lane 7: negative control. (TIF) [file pone.0282499.s001.tif]

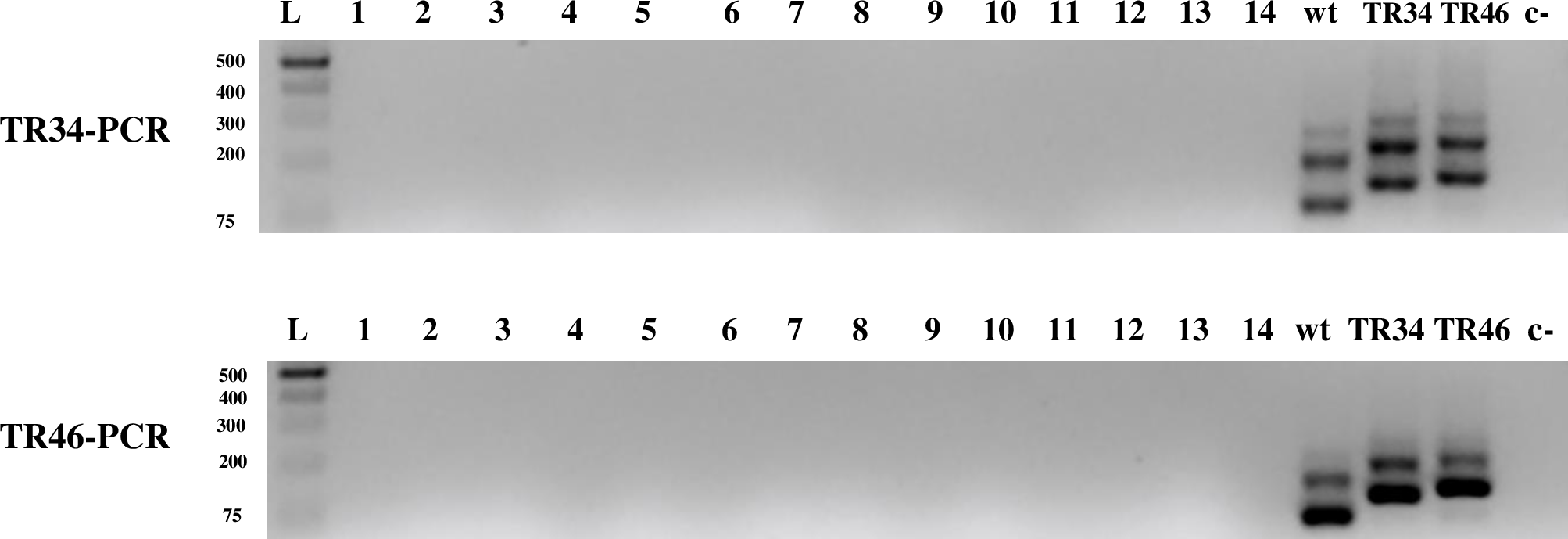

Supplement: S2 Fig — There was no amplification of DNA for any of the other microorganisms tested. There were amplification products of the wt, TR34, and TR46 A. fumigatus isolates. L: Ladder, lane 1: Actinomycete, 2: Bacillus sp., 3: Coccobacillus, 4: Candida sp., 5: Penicillium sp., 6: Chrysosporium sp., 7: Fusarium sp., 8: Paecilomyces sp., 9: Rhizopus sp., 10: Rhizomucor sp., 11: Mucor sp., 12: Cladosporium sp., 13: Aspergillus flavus, 14: Aspergillus niger, wt: wild-type A. fumigatus isolate, TR34: A. fumigatus isolate with the 34-nucleotide tandem repeat, TR46: A. fumigatus isolate with the 46-nucleotide tandem repeat. (TIF) [file pone.0282499.s002.tif]

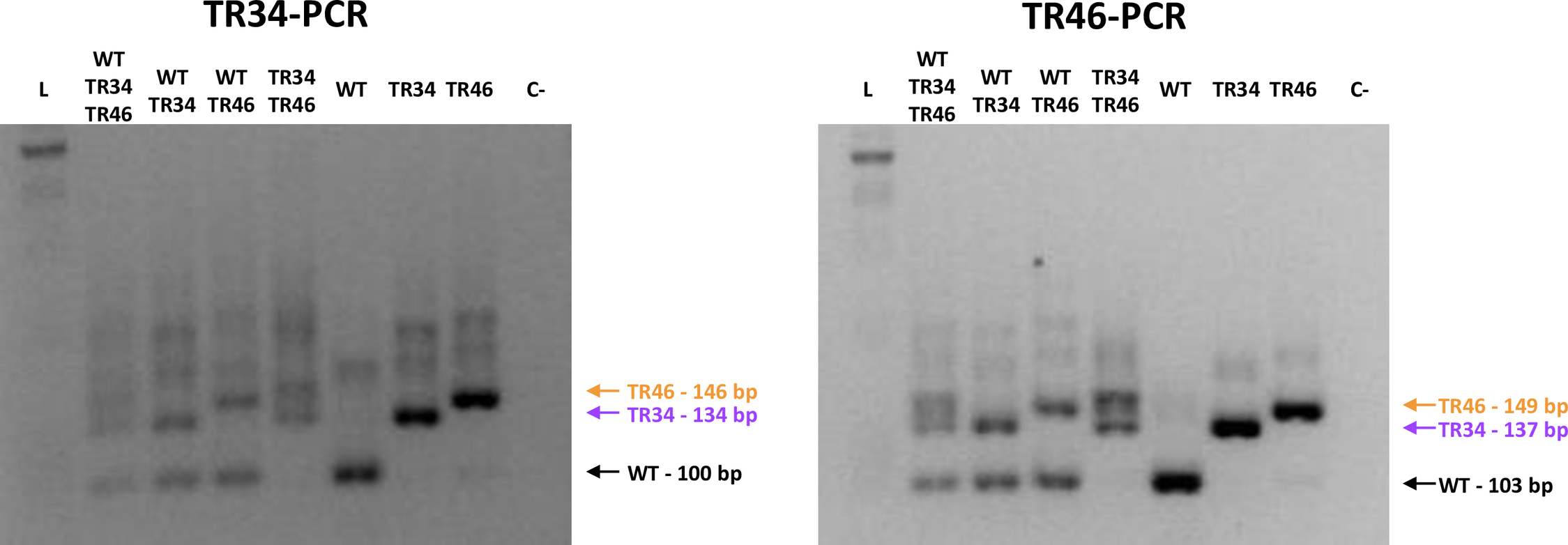

Supplement: S3 Fig — Both TR34-PCR and TR46-PCR amplify all alleles in mixtures. Here we mixed DNA from Aspergillus fumigatus controls with wt, TR34 and TR46 alleles in different combinations. DNA was mixed in equal proportions with final DNA concentrations of 0.2 ng/μl. It is possible to detect and distinguish the three alleles in various mixtures in a 4% agarose gel by comparing the fragment sizes in the mixtures with the fragments from the samples with single alleles. The bands in mixtures are fainter because the alleles are represented by either half or one third as much DNA as the single isolate samples in the template DNA used for PCR. (TIF) [file pone.0282499.s003.tif]

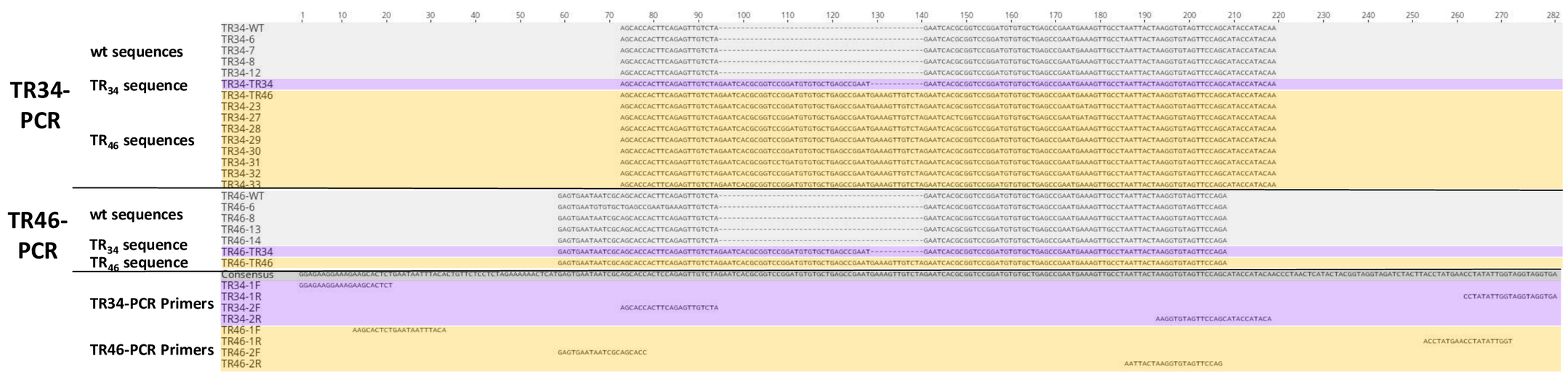

Supplement: S4 Fig — The PCR products from the environmental samples in Athens and Watkinsville, GA were confirmed as having the wt or TR46 alleles. Nucleotide lengths of sequences for control isolate alleles corresponded with the fragment sizes on the agarose gels. (TIF) [file pone.0282499.s004.tif]

Fig. 1

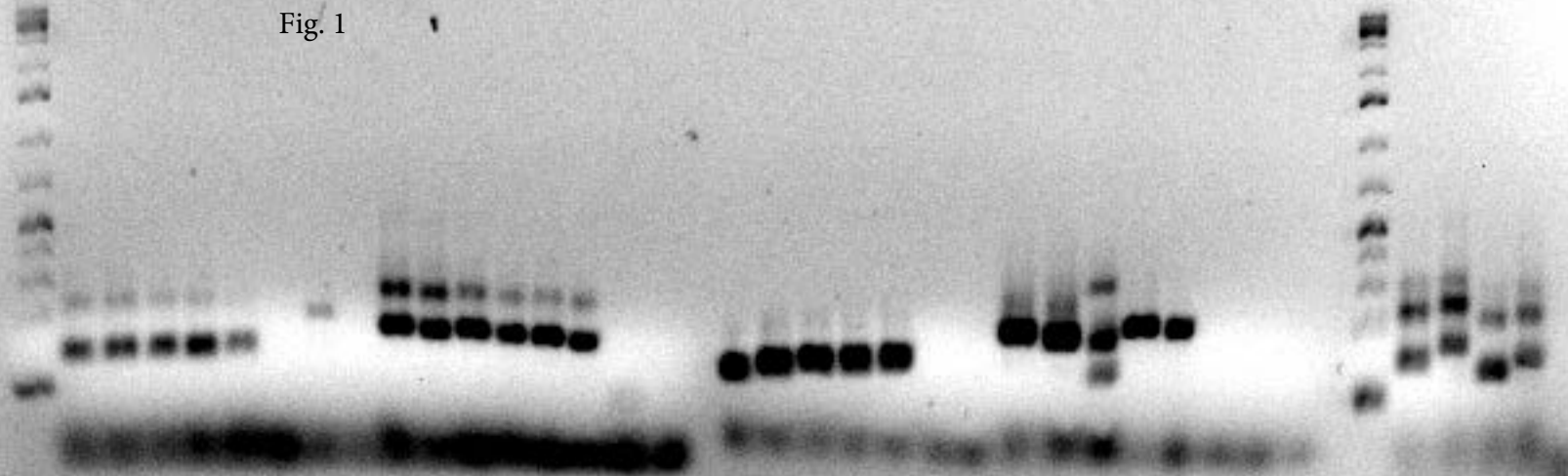

Fig. 2A

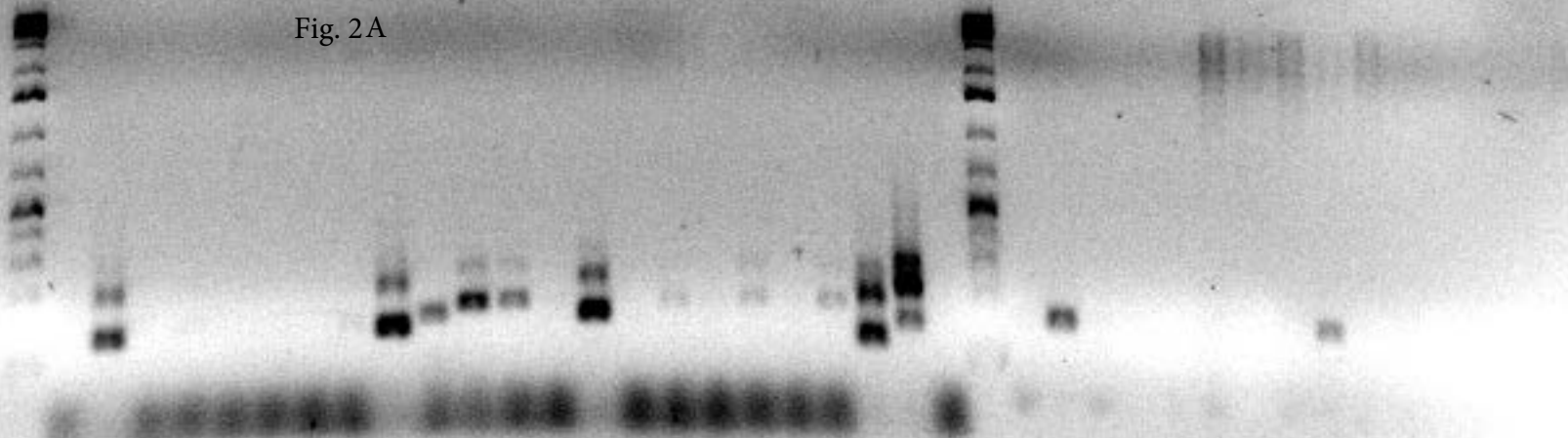

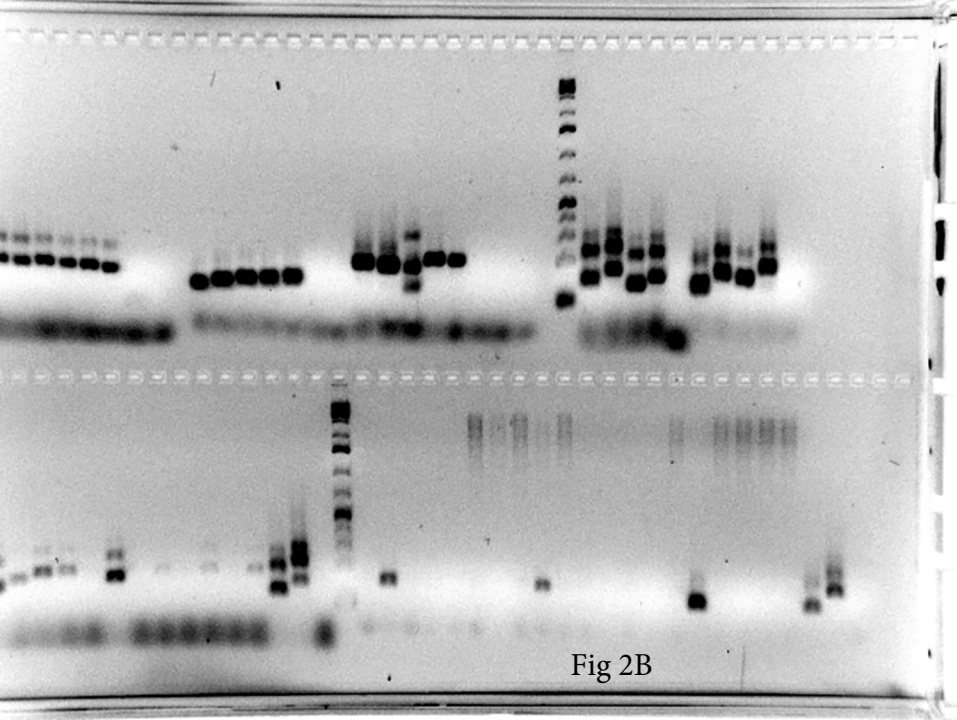

Fig 2B

Fig. 3

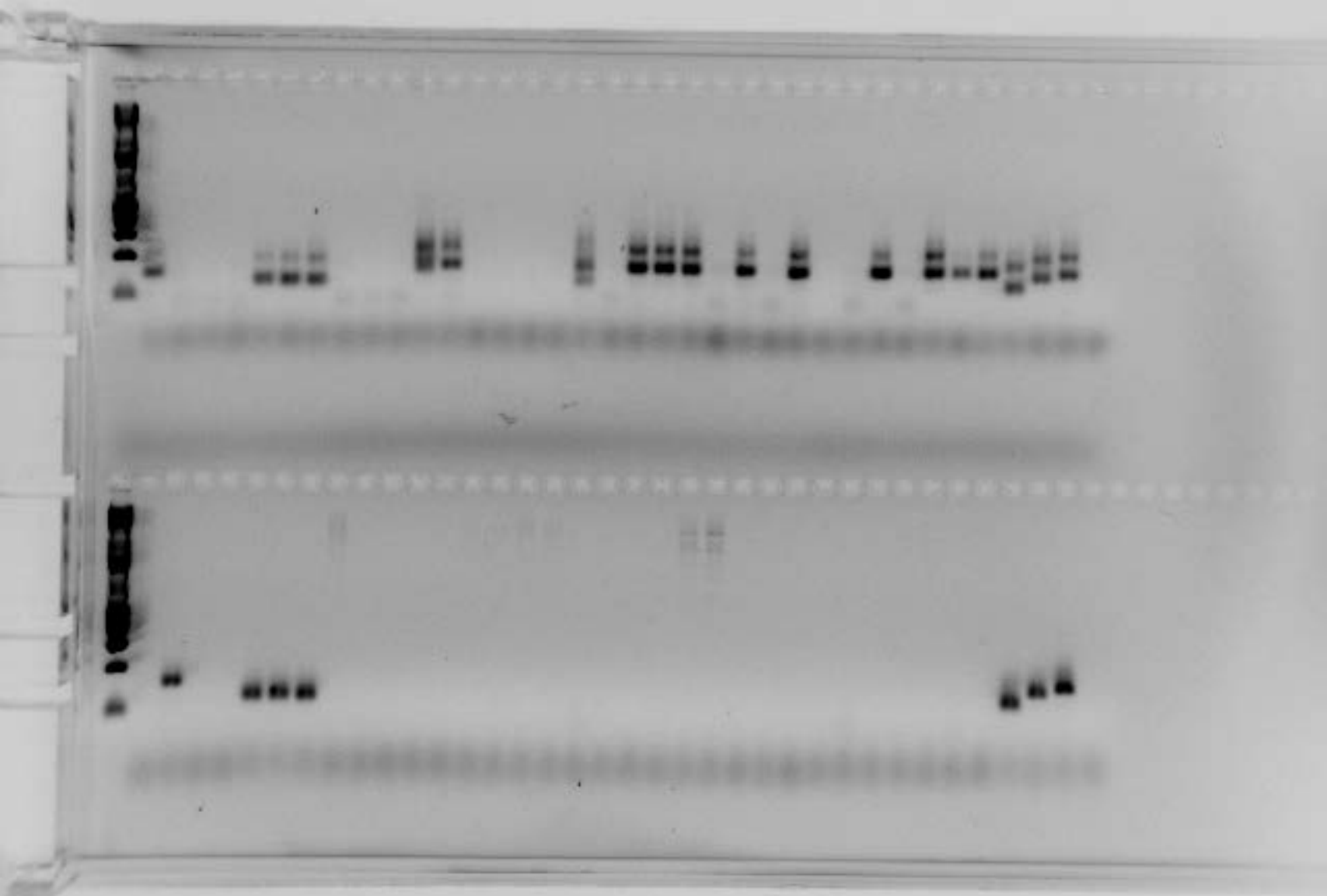

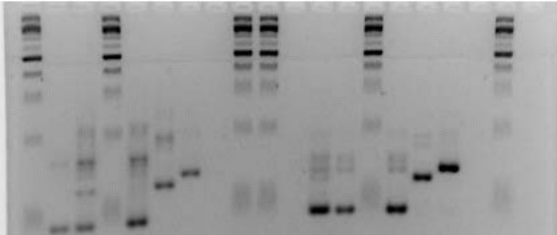

Fig. 4 left

Fig. 4 right

S1 Fig

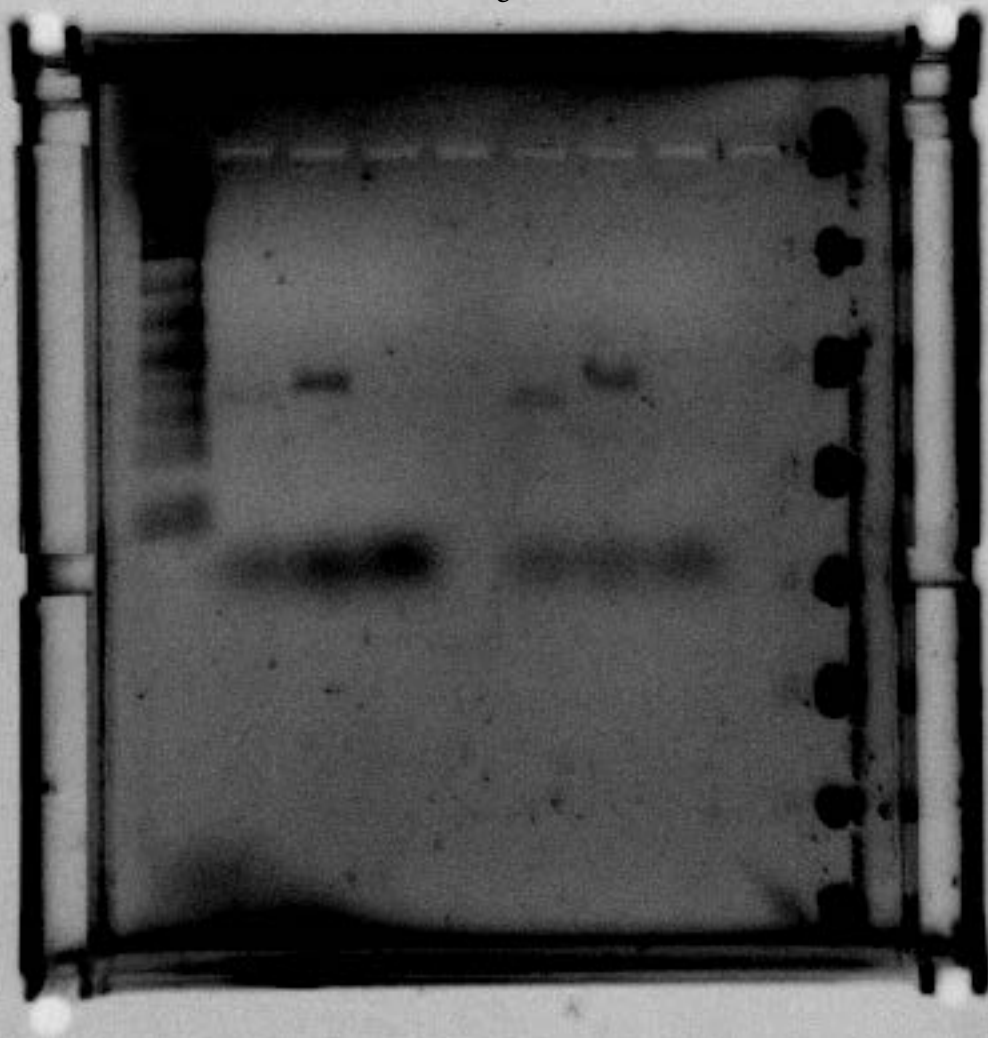

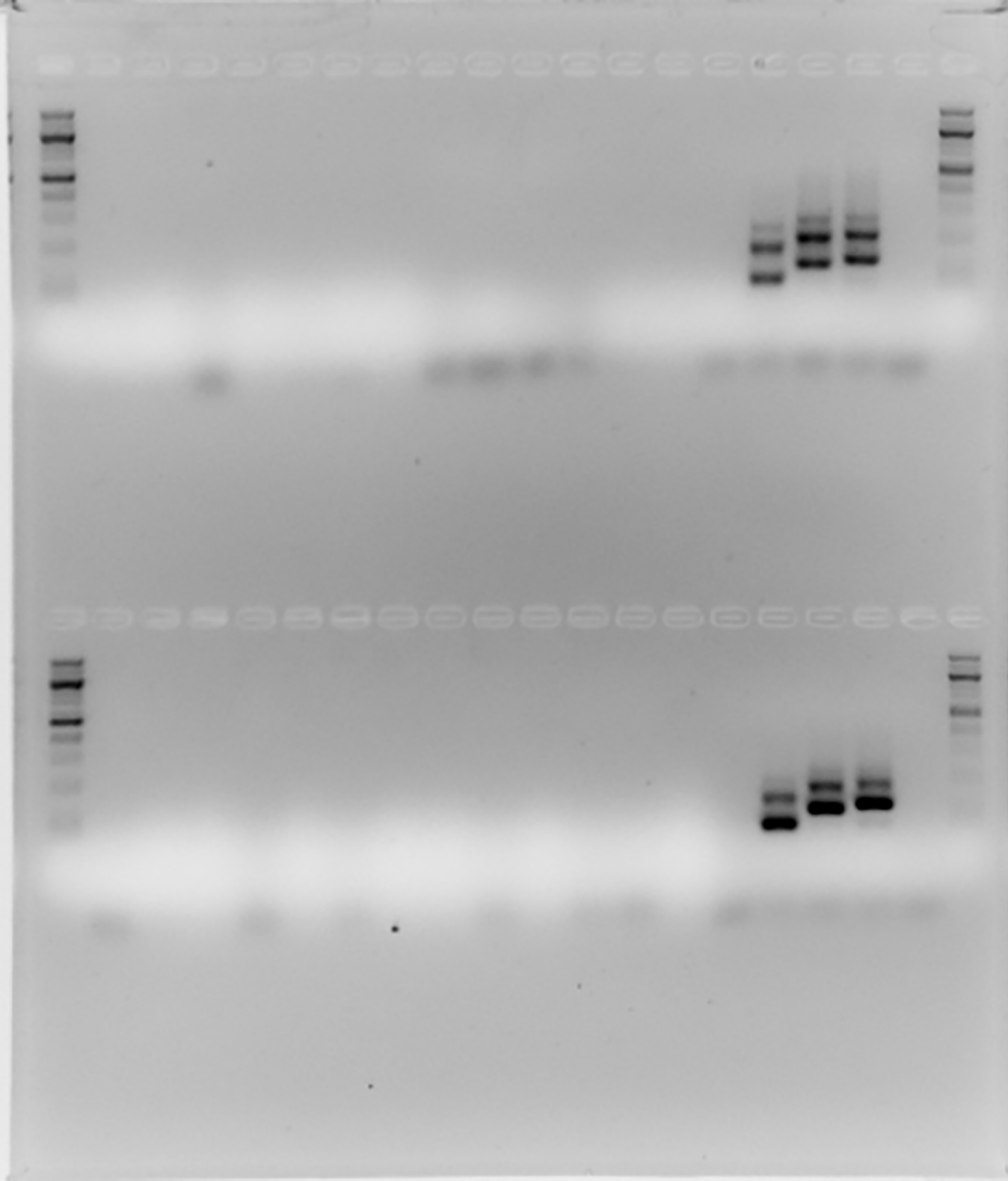

S2 Fig top

S2 Fig bottom

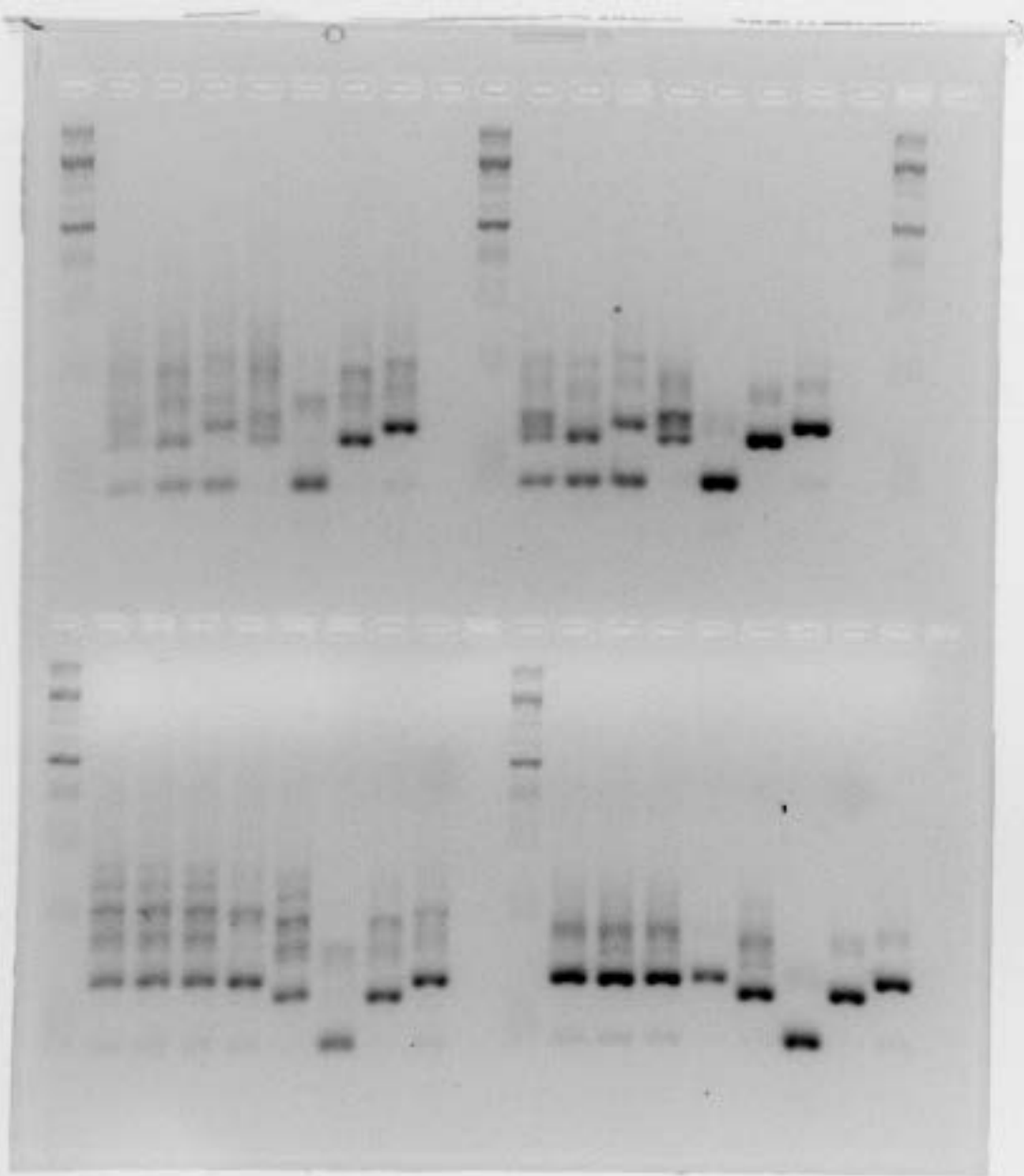

S3 Fig

(top part of gel is  
figure)

Supplement: S1 Raw images — (PDF) [file pone.0282499.s005.pdf]
